# Structure and Stability of Phospholipid-Based Microbubbles Studied Using a Spin Probe

**DOI:** 10.1021/acs.langmuir.5c03669

**Published:** 2025-10-17

**Authors:** Lauren E. Jarocha, Jason E. Streeter, Sherwood Ivan Weaver, Andrew McHorse, Natalia V. Lebedeva, Paul A. Dayton, Malcolm D. E. Forbes

**Affiliations:** † Department of Chemistry, 1888 University of North Carolina Chapel Hill, Chapel Hill, North Carolina 27599, United States; ‡ Department of Chemistry, 3628 Furman University Greenville, Greenville, South Carolina 29613, United States; § Lampe Joint Department of Biomedical Engineering, 2456 University of North Carolina at Chapel Hill, Chapel Hill, North Carolina 27599, United States; ∥ Lampe Joint Department of Biomedical Engineering, North Carolina State University, Raleigh, North Carolina 27695, United States; ⊥ Center for Photochemical Sciences, Department of Chemistry, Bowling Green State University Bowling Green, Ohio 43403, United States

## Abstract

Steady-state electron paramagnetic resonance (EPR) spectroscopy is used to investigate the structure and stability of surfactant microbubbles made from distearoylphosphatidylcholine (DSPC) phospholipids and a polymeric stabilizer. The spin probe doxyl-5-stearic acid (5DSA) was incorporated into the phospholipid monolayer at an overall concentration of 3 × 10^–7^ M. The bubbles were characterized by optical microscopy and found to range in diameter from 0.6 to 10 μm. The EPR spectrum of the spin probe at room temperature exhibited slow motion and ordering. This behavior was simulated using the microscopic order–macroscopic disorder (MOMD) model through the EasySpin software package. During the course of 12 h in the EPR sample tube, a sharper fast-motion nitroxide spectrum appeared, indicating degradation of the bubbles over time. This is attributed to the typical process of microbubble degradation following gas exchange and interactions between the sample and capillary walls, leading to bubble collapse and the formation of a liquid phase with 5DSA incorporated into liposomes, micelles, or free molecules.

## Graphical abstract

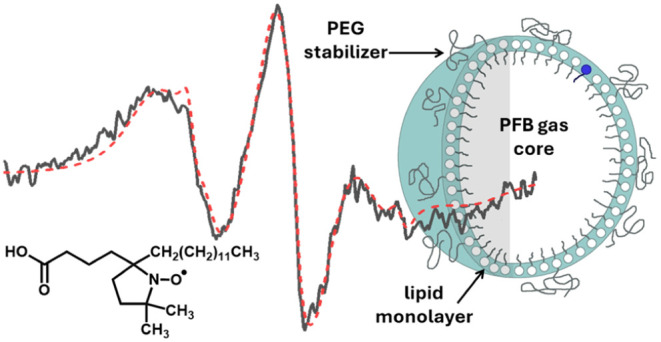

## Introduction

Surfactant-based nano- and microbubbles are unique and highly useful colloidal structures with applications in diverse fields such as biomedical imaging,^1–3^ drug delivery,^4,5^ and fossil fuel extraction.^6^ Microbubbles have a gas-filled core surrounded by a lipid monolayer that determines the bubbles’ properties. A key feature of these structures is their compressibility, which allows for an acoustic response in the 1–15 MHz region. While microscopy and rheological methods have provided much information about the macroscopic behavior of bubbles, only a few studies have been carried out to understand the structure and stability of microbubbles at the molecular level.^7–9^ This is surprising, as the bubble structure provides an opportunity to study the properties of lipid monolayers with a range of conventional spectroscopies that are not usually considered amenable to the study of air–water or solid–water interfaces.

Line shape analysis of the electron paramagnetic resonance (EPR) spectra of nitroxide spin probes has been used extensively to investigate various colloidal heterogeneous structures, including micelles, vesicles, and lamellar phases in surfactant mixtures,^10–15^ but to date, this technique has not been applied to bubbles or foams on the micro- or nanoscale. Similarly, spin probes have been applied to the study of lipid monolayers in only a limited capacity, such as through oil-in-water emulsions and phospholipid reverse micelles,^16,17^ but there has been no application of the technique to the study of lipid organization at a liquid–gas interface. In this article, we report the results of steady-state EPR experiments on polymer-stabilized, phospholipid-based microbubbles with a nitroxide radical spin probe (doxyl-5-stearic acid) incorporated into their outer wall (Chart [Fig chart1]).

**1 chart1:**
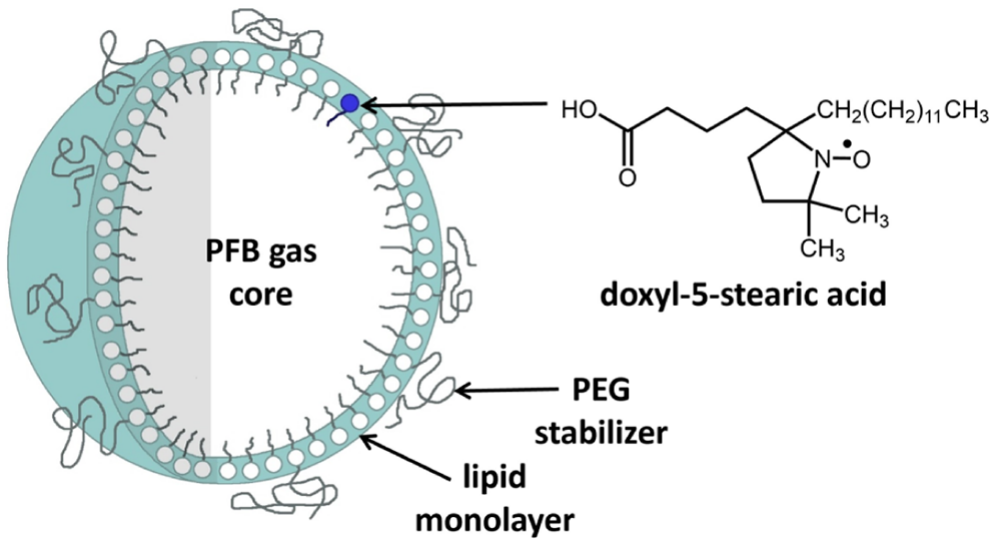
Polymer-Stabilized, Phospholipid-Based Microbubbles with a Stable Nitroxide Radical Spin Probe (Doxyl-5-stearic acid) Incorporated into Their Outer Wall; PFB = Perfluorobutane

The EPR spectrum of the spin probe is expected to be highly sensitive to the mobility and orientation effects in the hydrophobic regions of the bubble. The goal of this study is to better understand the mobility, order, and stability of these novel colloidal structures at the molecular level.

## Experimental Section

### Bubble Preparation

A lipid suspension containing 90 mol % distearoylphosphatidylcholine (DSPC) and 10 mol % 1,2-distearoyl-*sn*-glycero-3-phosphoethanolamine-PEG40-stearate (DSPE-S) (Avanti Polar Lipids, Alabaster, AL), along with doxyl-5-stearic acid (5DSA, Sigma-Aldrich, St. Louis, MO), was prepared in phosphate-buffered saline (PBS; pH 7.4) by first dissolving the lipid and spin probe in chloroform. The solvent was evaporated under a steady stream of nitrogen, and the surfactants were redispersed in 100 mL of PBS by heating the lipid solution to 60 °C within an ultrasonic cleaning device (Model 450A, Branson Ultrasonics, Danbury, CT). The final lipid concentration in the stock solution was 1.5 mg/mL and 3 × 10^–7^ M doxyl-5-stearic acid. A 1 mL aliquot of the lipid stock solution was added to a 3 mL vial. The headspace of the vial was gas-exchanged with perfluorobutane (PFB) (FluoroMed, Round Rock, TX), and microbubbles were formed by mechanical agitation of the vials. The bubbles were washed several times with a PBS buffer solution to remove any excess spin probe from the solvent. This process also removed liposomes and smaller bubbles. The resultant bubble suspension was optically imaged by using a Zeiss Axioskop 2 microscope. Analysis of bubble size was performed using ImageJ.JS.^18^

### EPR Spectroscopy

All experiments were performed on a JEOL, USA, Inc. FA-100 X-band (9.5 GHz) instrument using 100 kHz field modulation. Samples were kept oxygen-free prior to transfer into a 0.5 mm inner diameter quartz capillary. Capillaries were sealed with Critoseal and placed in the center of a TE_011_ cylindrical resonator. For a single scan, the sweep time was 4 or 8 min, and the time constant was 0.3 or 1 s, respectively. Scans were repeated up to 25 times and accumulated to improve the signal-to-noise ratio. The modulation amplitude was 5 G for broad signals and reduced to 2 G when sharper line widths were detected.

### Spectral Fitting

Data were fit using EasySpin’s esfit function, with the appropriate program (garlic or chili) for the motional regime of the data.^19^ Values for the g- and A-tensor of the 5DSA spin probe were taken from the literature.^20,21^ Fitting parameters included rotational correlation time, peak-to-peak line width, and the A_*zz*_ component of the hyperfine tensor. For data that showed molecular ordering, a fixed value of diffusion tilt angle, β_D_, of 34° was employed, and the ordering potential, C_20_, was included as a fitting parameter.

## Results and Discussion

Figure 1 shows an optical micrograph of the bubbles constructed from a 90:10 mol ratio DSPC:DSPE-S suspension, as described in the experimental section. The role of DSPE-S, which contains a polyethylene glycol (PEG) unit on one end, is to act as a stabilizer for the bubble structure.^22^ Such bubbles are known to survive for a minimum of several hours once exposed to the atmosphere but remain stable for much longer periods when stored under PFB.^23^ The bubbles range in diameter from about 0.6 to 10 μm, which is approximately the size of mammalian tissue cells. Figure S1 shows the size distribution of the bubbles based on this image. No further attempts to improve the polydispersity were made for this preliminary study.

**Figure 1:**
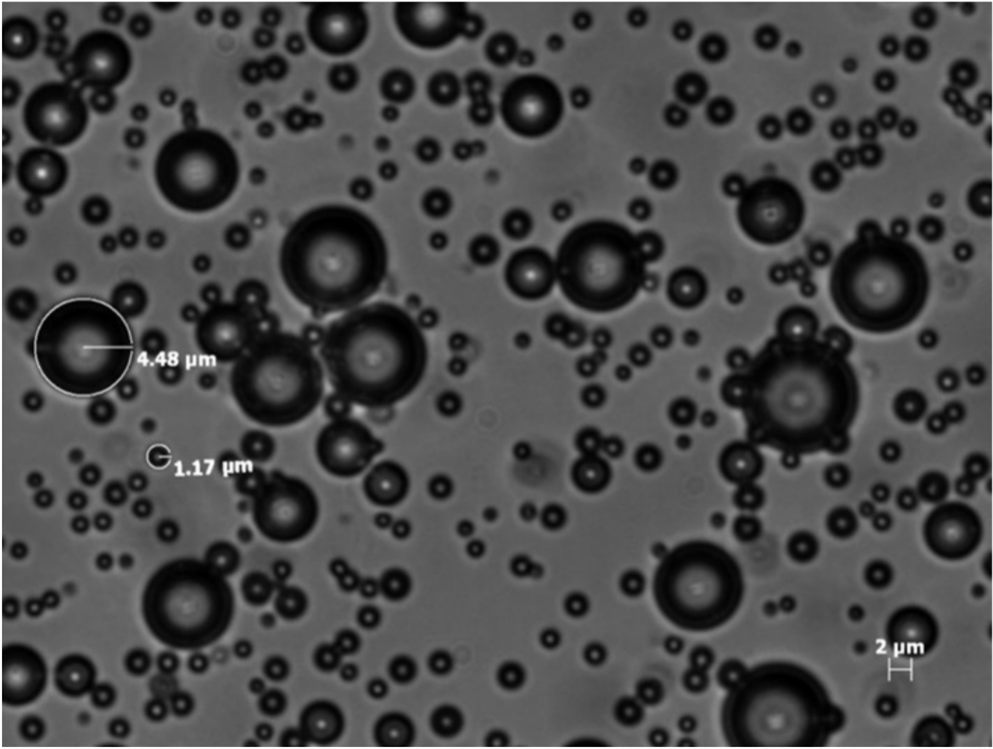
Optical micrograph of an aqueous suspension of DSPC:DSPE-S microbubbles.

Figure 2 shows the steady-state X-band EPR spectrum acquired for a suspension of DSPC:DSPE-S microbubbles in aqueous solution immediately after bubble preparation (black line). The spectrum is broad and anisotropic, which is characteristic of the slow-motion regime for the rotation of the nitroxide spin probes.^24,25^ There is strong evidence suggesting that there may be more than one component in the spectrum, indicative of spin probes occupying more than one motional regime. The bubble composition suggests two potential locations for incorporation of the probe: within the lipid layer or interacting with the PEG stabilizer on the surface. In addition, the micrograph in Figure 1 indicates a wide range of bubble sizes. Properties of the environment in which the spin probe incorporates (i.e., viscosity, hydration, ordering) may be a function of bubble size, though there is increasing evidence that even in monodisperse preparations, some physical properties of lipid microbubbles still vary widely.^26,27^ Given this, a multicomponent EPR spectrum is not surprising. With such broad lines, any fit may be easily overparameterized. To extract meaningful data, baseline values for as many parameters as possible were established from the control measurements.

**Figure 2:**
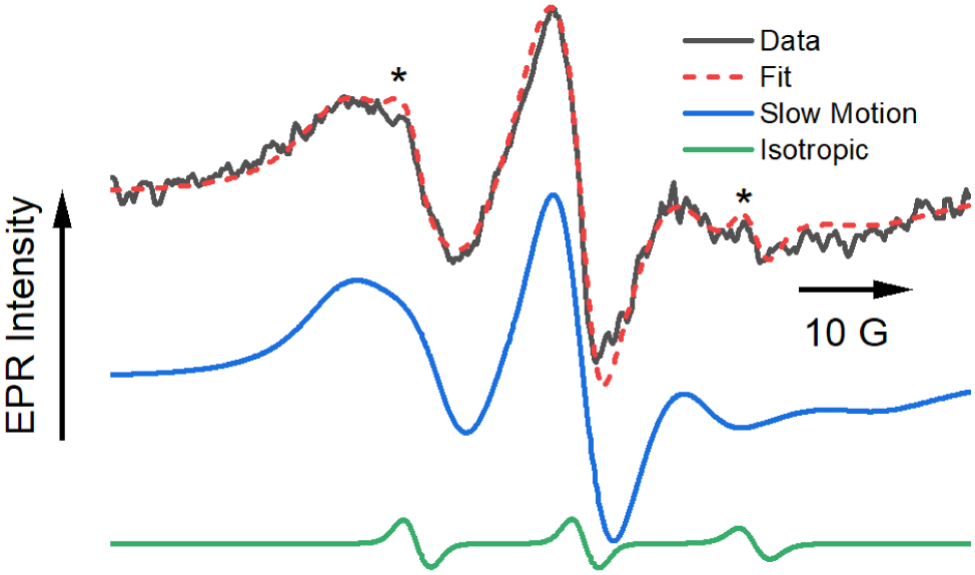
X-band EPR spectrum of an aqueous suspension of DSPC:DSPE-S microbubbles doped with doxyl-5-stearic acid (black), along with a computer simulation using the Freed MOMD model (red).^19,20^ The spectrum is a single scan to avoid complications arising from bubble degradation. The peaks marked with asterisk*s* arise from the minor isotropic component. The slow motion (blue) and isotropic (green) contributions to the fit are shown separately. See text for additional details.

Figure 3 shows the EPR spectrum and the corresponding fit obtained using EasySpin’s garlic function for isotropic rotational motion of 5DSA in the PBS buffer solution in the absence of any lipids. The parameters obtained from this and all subsequent fits to the data are summarized in Table 1. The value of the isotropic rotational correlation time, τ_c_, was 0.29 ns. The *A*_*zz*_ component of the hyperfine tensor may be used as an indication of the local hydration environment in the vicinity of the probe; its value was 35.6 G. The fit also includes a peak-to-peak Gaussian line width of 2.7 G. The line width parameter is often correlated with other variables of interest, most notably the rotational correlation time. For all subsequent fits shown herein, this line width was held constant.

**Table 1:** Summary of Parameters Obtained from Fits to Experimental Data Is Given inFigures [Fig fig2]–[Fig fig3] [Fig fig4]

sample	τ_c_(ns)	τ_⊥_(ns)	τ_∥_(ns)	*A*_zz_(G)	*C*_20_
buffer	0.29	–	–	35.6	–
liposomes	–	21.3	0.46	32.0	2.44
microbubbles	–	4.4	0.49	35.6	2.72

**Figure 3:**
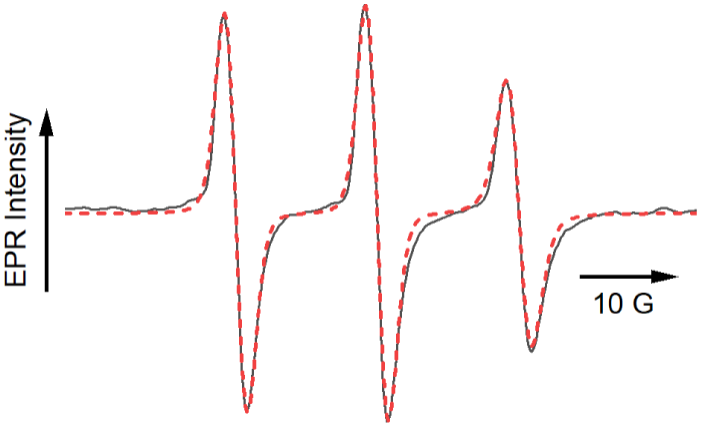
EPR spectrum of 5-doxyl stearic acid (black) at a concentration of 3.0 × 10^–7^ M in a solution of PBS buffer at pH 7.4. Data were fit with EasySpin (red). Parameters obtained from the fit may be found in Table 1.

Omitting the agitation stage in the preparation procedure prevents the formation of bubbles, providing a benchmark for the EPR spectrum and properties of nanostructures formed from the spontaneous self-assembly of lipids in solution. Figure 4 shows the experimental data and fit of the EPR spectrum of 5DSA incorporated into a solution of 1.5 mg/mL of 90:10 mol ratio DSPC:DSPE-S without agitation. In this case, the EPR spectrum is clearly multicomponent. The 5DSA spin probe can be solubilized in the PBS buffer; therefore, it is likely that the 5DSA will partition between the solution and any lipid-based structures that form. Such partitioning would lead to a superposition of probes with different degrees of ordering and very different rotational correlation times.

**Figure 4:**
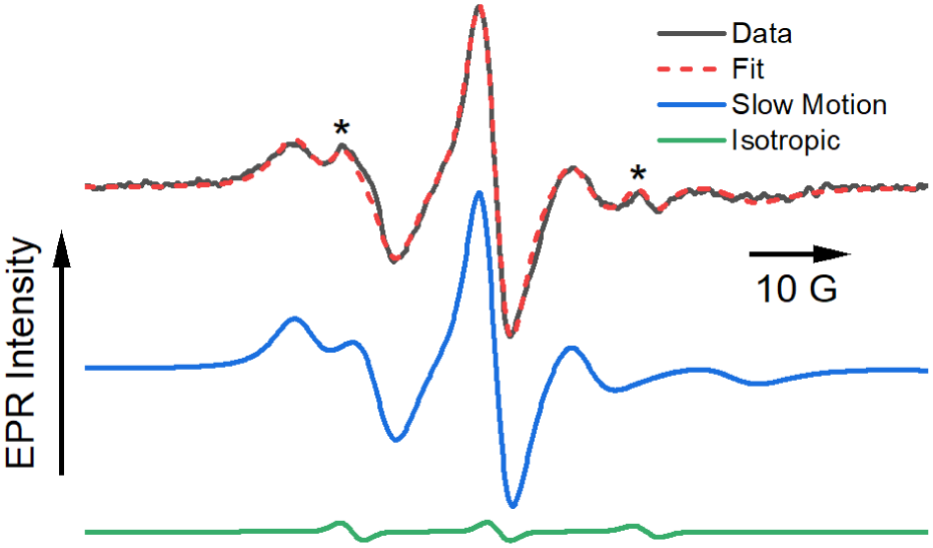
X-band EPR spectrum of the same lipid/probe system as above except that the sample was gas-exchanged with PFB but not agitated prior to the experiment. The black line represents the data, while the dotted red line represents the fits obtained from EasySpin in the regime of slow rotational motion of the probe. The peak marked with the asterisks arises from the minor isotropic component. The slow motion (blue) and isotropic­(green) contributions to the fit are shown separately.

A simulation of the lipid spectrum (Figure 4) can be achieved with two components: a fast-motion isotropic spectrum of 5DSA and a second component in the slow-motion regime. The properties of the slow-motion component were fit using EasySpin’s chili function and the microscopic order macroscopic disorder (MOMD) model developed by Freed and coworkers.^28,29^ Briefly, this model assumes the existence of domains of local ordering, described by an orienting potential normal to the microbubble surface. The axially symmetric spin probe (Scheme 1, left side) will adopt a preferred orientation in the bubble relative to the local ordering of the DSPC and DSPE-S lipids. The degree of ordering of the probe can be described by the axial order parameter, *S*_20_, with a value of 1 corresponding to perfect alignment of the probe with the director potential.^30^ While ordered in the microscopic environment of the bubbles, the director frame of reference for the local ordering potential experienced by any individual probe molecule is isotropically distributed relative to the axis of the applied magnetic field of the EPR spectrometer; therefore, there is no observable macroscopic order (right side of Scheme 1) within the sample. The MOMD model provides a means to simulate individual ordered spectra on the microscopic level but then integrates over the macroscopic distribution to obtain the spectrum of the ensemble. This model has found wide utility in fitting intermediate to slow-motion spectra of nitroxide spin probes in a variety of heterogeneous media.^31–35^ Comparable fits to the data in Figure 4, but excluding the ordering parameter of the MOMD model, can be found in Figure S2.

**Scheme 1:**
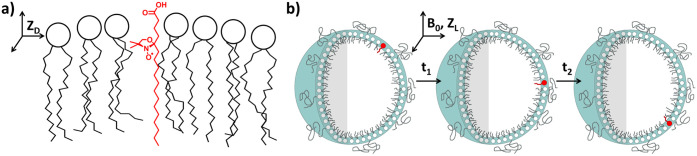
Microscopic Order Macroscopic Disorder (MOMD) Model as Applied to Microbubbles[Fn sch1-fn1]

a
The doxyl-5 spin probe is highly ordered in the surfactant monolayer (microscopic order, a) but is distributed randomly about the sphere (macroscopic disorder, b). The model developed in references 19 and 20 provides a means to simulate the EPR spectra contained herein and assess the degree of order in this system.

The peaks in Figure 4 marked with asterisks result from a spin probe experiencing a low degree of order and relatively fast rotational motion—consistent with a small amount of the spin probe located in the PBS buffer. Parameters for this component were fixed according to the results of the fit shown in Figure 3; only its weight was varied. The broad and anisotropic line shape of the high- and low-field lines of the remaining component suggests that the spin probe primarily incorporates into an environment with a relatively high degree of ordering and a slow rotational correlation time. In the slow-motion regime, the rotational motion of the axially symmetric 5DSA is appropriately considered as anisotropic, with two components—τ_⊥_for rotational motion on the degenerate axes and τ_∥_ for the unique rotational axis. Values obtained from the fit shown in Figure 4 are as follows: τ_⊥_ = 21.3 ns, τ_∥_ = 0.46 ns, *A_zz_* = 32.0 G, *C*_20_ = 2.44. The quality of the fits is improved by allowing for a diffusion tilt angle, β_D_, of 34° between the primary rotational axes of the molecule and the magnetic axes of symmetry of the g-tensor. This suggests that the alkyl tail of the probe adopts a bent conformation.^36,37^ The weights of the isotropic and anisotropic components were 0.03 and 0.97, respectively. These parameters are generally in line with literature values for the behavior of doxyl spin probes in vesicles,^38,39^ though the perpendicular rotational correlation time is about an order of magnitude slower and the ordering potential parameter slightly higher than typical. Most EPR studies of lipids are conducted above the melting temperature, *T*_m_, of the gel-to-liquid crystal phase transition of the lipids,^40^ though studies using spin-labeled phosphatidylcholines that compare rotational motion in the gel and liquid phases indicate a change in the rotational diffusion tensor of approximately one to two orders of magnitude associated with this phase transition.^29,41^ The measurements reported here were carried out at room temperature, well below the *T*_m_ values of 55 °C of DSPC and 75 °C of DSPE; therefore, a slower rotational correlation time is expected. The value of *C*_20_ obtained from the fits corresponds to an order parameter, *S*_20_, of 0.52, indicating a preferential, but incomplete, alignment of the spin probe normal to the surface of the bubble. The *A*_*zz*_ value is smaller for the lipid solutions relative to that of the buffer, consistent with the incorporation of the probe into the more hydrophobic environment of a liposome.

With these parameters as a benchmark, it is possible to return to the simulation of the spectrum of 5DSA incorporated into the bubbles (Figure 2, dashed red line). Fitting of this data allows for a qualitative comparison of the properties of the microbubbles, including polarity, diffusional motion, and molecular order experienced by molecules incorporated in these structures, in contrast to liposomes (Figure 4) and free solution (Figure 3). The simulation was carried out with two components: one that is isotropic and another using the MOMD model.^28,29^ Parameters for the isotropic component were fixed according to results obtained from 5DSA in the buffer (Figure 3). Simulation of the slow-motion component returned values of τ_⊥_ = 4.4 ns, τ_∥_=0.49 ns, *A*_*zz*_ = 35.6 G, and *C*_20_ = 2.72. Heisenberg spin exchange was included in the fit, with a resulting value of 10.48 MHz. The normalized relative weight of the isotropic component was 0.014. Comparable fits to the data in Figure 2, but excluding the ordering parameter of the MOMD model (Figure S3) or Heisenberg spin exchange (Figure S4), can be found in Supporting Information.

The order parameter for the probe incorporated into the bubbles is comparable to that in the lipid solution (0.56 vs 0.52). This degree of ordering is consistent with that observed in other supramolecular surfactant aggregates, including vesicles^20,21,40^ and viscous micelles.^27–30,38,42^ It is known from the literature that doxyl stearic acid spin probes incorporate into lipid structures.^43–45^ A study of 5DSA incorporation into the micellar phase of L64, a triblock copolymer of poly­(ethylene oxide) (PEG) and poly­(propylene oxide) (PPO) that forms nonionic micelles in aqueous solution, revealed very low values for the hyperfine interaction, consistent with 5DSA partitioning strongly into the hydrophobic PPO regions of the structure.^46^ An order parameter comparable to that reported here is required to fit data for 5DSA when NaOH is added, which facilitates a transition to a lamellar phase of the polymer. Taken together, these observations suggest that the probe likely incorporates into the organized lipid layer of the bubbles, as opposed to the PEG-stabilized surface layer. This is reflected in Chart [Fig chart1]. The value of the rotational correlation time is faster in the lipid spectrum than in the bubbles. Rotational anisotropy, measured as the ratio between the perpendicular and parallel rotational correlation times, τ_⊥_/τ_∥_, is smaller in the bubbles than observed in the liposomes. This indicates that the probe has more conformational freedom in the microbubble structure relative to the lipid solution, primarily due to a decrease in τ_⊥_. This is consistent with the difference in structure; lipids in the bubble form primarily a monolayer structure at the gas–liquid interface, whereas lipids in vesicles form bilayers. The motional freedom of small molecules incorporated into lipid monolayers depends on the surface pressure of the monolayer, which is a tunable parameter in studies by Langmuir–Blodgett troughs but cannot be easily controlled in lipid microbubbles.^47,48^ Based on EPR studies of surfactant and lipid monolayers formed at an oil–water interface,^17,49^ a faster rotational correlation time of the spin probe is expected for monolayer structures, consistent with the data reported here. *A*_*zz*_ is closer to the value observed in the PBS buffer (35.6 G) than in the lipid solution (32.0 G). 5DSA is generally considered a reporter for the local environment near the headgroups of surfactants and lipid assemblies. The higher value of *A*_*zz*_ observed in microbubbles compared to liposomes and the faster rotational correlation time suggest that the environment within the lipid layer of the bubbles allows for higher local hydration at the site of the nitroxide and more motional freedom, consistent with the lipids in the monolayer being less tightly packed than those in the bilayer.

The inclusion of Heisenberg spin exchange in the simulations deserves additional comment. Spin probe studies of surfactant assemblies are designed to keep probe concentrations low—ideally such that there is no more than one molecule incorporated into each structure. This works well for studies of micelles, where aggregation numbers are low. However, with vesicles, the aggregation number is significantly higher (>40,000 molecules/vesicle), resulting in a trade-off between the detection limit of the spectrometer and the occupation number of the spin probe in each structure. The aggregation number for the microbubbles, which are much larger in size, will be commensurately larger (Table S1). Each bubble will contain a significant number of spin probes. While it is technically possible that probe molecules may interact with one another within a bubble structure, or even across an interface where bubbles come in contact, lipid-to-probe ratios were kept within generally acceptable ranges for EPR studies (approximately 0.02 mol %). However, small amounts of Heisenberg spin exchange are not unexpected—and can even be leveraged to characterize heterogeneous structures formed from lipids. Many processes occurring in lipid membranes depend on the translational diffusion of molecules through the structure. Collisional frequencies of spin probes have been used to measure lateral diffusion through pulsed EPR techniques like ELDOR and saturation recovery EPR, including with doxyl-stearic acid probes in liposomes.^50^

EPR methods used to examine spin–spin interactions require a high concentration (>2 mol % generally, and 0.1–0.5% for lipid analogue labels) to ensure significant exchange-induced line broadening.^50^ At their highest, the spin probe concentrations in this study are an order of magnitude smaller (Table S1). However, the lateral diffusion coefficient of lipid monolayers formed at an air–water interface is found to be faster (15–35 μm^2^/s) than in comparable bilayers (6–7 μm^2^/s), with greater variability as a function of lipid identity.^51^ This is consistent with our results that showed increased conformational flexibility via EPR, as evidenced by the faster τ_⊥_. This would also lead to an increase in the diffusive encounters of spin probes in these structures, increasing the likelihood of spin–spin exchange contributions to the spectral line shape. However, this is likely insufficient to fully account for the observed effect, as the exchange rate from the simulation is comparable to what is observed for concentrations of paramagnetic species closer to 10–30 mM in solution.^52,53^

Heisenberg spin exchange fundamentally represents a source of inhomogeneous broadening in an EPR spectrum. Characterization of microbubble properties via fluorescence lifetime imaging reveals that microbubbles formed of pure DSPC have heterogeneous microenvironments within the lipid layer of the structure, with rotational correlation times of the fluorescence probes that vary significantly.^7,9,54^ This was attributed to local structural variations in the lipid shell. Phospholipid monolayers may form liquid crystalline and polymorphous domains with different tail group orientations.^55^ Evidence from fluorescence measurements also indicates the formation of microdomains with a bilayer structure.^9,56,57^ Other studies of the shell properties reveal a range of values as well; for example, shell viscosity varies by a factor of at least 3 and up to an order of magnitude,^27,58^ with no apparent correlation to bubble size. This variability is attributed to the formation of microdomains within the bubble shell, the distribution of which is unique to each bubble. Therefore, within an individual microbubble, one can expect variations in the organization and packing of the tails of the phospholipids, as well as the possible presence of lamellar domains and, consequently, changes to local hydration or motional freedom, all of which would influence the line shape of the EPR spectrum.

The data in Figure 2 can be appropriately considered as a superposition of spectra for 5DSA with a range of values of *A*_*zz*_, τ_⊥_, τ_∥_, and *C*_20_. Figure S5 shows the simulation of the EPR spectrum using the parameters obtained from the fit of the data in Figure 2 in the absence of spin exchange and over a range of values for these parameters. The necessity to include spin exchange to achieve appropriate fits is, at least in part, a consequence of the need for inhomogeneous broadening of the spectrum, though local high concentrations of the probe leading to significant spin–spin interactions may occur in some of the lipid phases of the bubble shell.

A final noteworthy observation during these experiments was the detection, over the course of several hours, of a new fast-motion component in the signal in the EPR spectrum of the original microbubble preparation. Figure 5 shows EPR data acquired by monitoring and averaging the EPR spectrum over 12 h. The asterisks indicate two fast-motion peaks that grew over time, and there is presumably a third transition underneath the broad central line in the spectrum. The stability of microbubbles has been the subject of several literature reports,^57,59,60^ which show that monolayer collapse occurs in discrete stages. Microbubbles exhibit a wrinkle-to-smooth change in their surface under optical microscopy, during which excess lipid is expelled from the lipid monolayer.^61^ This process of collapse is accompanied by changes in the composition and structure of the lipid shell. Kwan and Borden followed the kinetics of the decomposition of several lipid formulations stabilized by PEG-40-stearate or PEG-modified DSPE.^22^ They found that for rigid lipid encapsulations, once dissolution begins, the process will continue until the microbubbles stabilize at a uniform size. For mixtures of DSPC:DSPE-PEG_2000_ that were gas-exchanged, this process took several minutes. Once the bubbles reached a size of 1–2 μm, the majority remained stable. We attribute the observations in Figure 5 to this degradation process, which is most likely induced by a combination of collisions or interactions with the walls of the quartz sample tube and the escape of the PFB gas from the bubble core. The relative increase in the isotropic contribution to the spectrum, marked with asterisks in the figure, can be attributed to the 5DSA spin probe being expelled into the buffer solution with the lipids during bubble collapse, where the spin probe is free to tumble. The time scale for the destabilization process as observed by EPR is in line with observations of similar bubble preparations using different physical methods.^23^ While there was no obvious macroscopic indication of bubble degradation from visual inspection of the solution over time, the EPR data clearly indicate that at the molecular level, rather extensive degradation is occurring.

**Figure 5:**
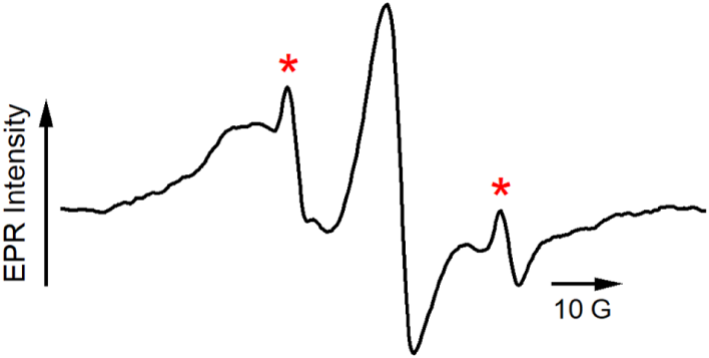
X-band steady-state EPR spectrum of the same sample shown in Figure 1 after 12 h. The red asterisks indicate peaks from a fast motion component that have grown in over time.

## Conclusions

This work demonstrates that EPR can be successfully employed for the investigation of gas-filled, lipid-based microbubbles, which can be leveraged to better understand the loading and diffusion of small molecules within these structures. The incorporation of the spin probe is straightforward, and the microscopy results suggest minimal perturbation of the bubble structure. EPR spectroscopy reveals differences in molecular ordering and conformational freedom of the probe in the bubbles compared to free solution or lipid vesicles, which correlate with structural differences between the liquid–liquid and liquid–gas interfaces. The necessity of including a mechanism for inhomogeneous line broadening is attributed to inhomogeneity in the local environment in which the probe incorporates, which is most likely a consequence of the formation of local domains of lipids with different structures and physical properties in the bubble shell and the broad distribution of bubble sizes. EPR is also capable of detecting the degradation of the bubbles over several hours, providing additional information about the stability of these structures when exposed to ambient or other conditions. The rate of degradation of microbubbles affects their utility. For example, in medical applications, the dissolution or collapse of the bubbles limits their circulation time in the blood when used for drug delivery^57^ or reduces the photoacoustic response when employed as contrast agents for ultrasound imaging.^62^ The observation of changes to the EPR spectrum as a function of time represents a new method to monitor the degradation of bubble dissolution and shell collapse. This technique could be applied to monitor bubble stability under a wide range of conditions relevant to their application in medical diagnostics, drug delivery, and acoustic imaging.

## Supplementary Material



## Notes

Histogram showing distribution of bubble sizes, table summarizing spin probe loading as a function of liposome or bubble size, comparison of fits of EPR data without ordering potential or Heisenberg spin exchange, simulations showing the effects of key fitting parameters on EPR line shape (PDF)

## #

Department of Engineering, Loyola University of Chicago, Chicago, IL, 60660, United States

## ∇

National High Magnetic Field Laboratory, Department of Chemistry, Florida State University, Tallahassee, FL 32306, United States.

## ○

School of Medicine, Duke University, Durham, NC 27710, United States.

## ◆

Syngenta Crop Protection LLC, P.O. Box 18300, Greensboro, NC 27419, United States.
