# Supplementary material for: Structure and Stability of Phospholipid-Based Microbubbles Studied Using a Spin Probe
Source: Langmuir. 2025 Oct 17;41(42):28513–20. doi: 10.1021/acs.langmuir.5c03669 (PMC12573782; doi:10.1021/acs.langmuir.5c03669)
Supplement: Supplementary file 1 [file la5c03669_si_001.pdf]

Supporting Information

Structure and Stability of Phospholipid-based Microbubbles Studied Using a Spin Probe

Lauren E. Jarocha*,[a,b] Jason E. Streeter,[c,f] Sherwood Ivan Weaver[b,g], Andrew McHorse[b,h], Natalia V. Lebedeva,[a,i] Paul A. Dayton,[c,d] and Malcolm D. E. Forbes[a,e]

[a] *Department of Chemistry, University of North Carolina, Chapel Hill, NC 27599*

[b] *Department of Chemistry, Furman University, Greenville, SC 29613*

[c] *Lampe Joint Department of Biomedical Engineering, University of North Carolina at Chapel Hill, Chapel Hill, NC 27599 and*

[d] *Lampe Joint Department of Biomedical Engineering, North Carolina State University, Raleigh NC 27695*

[e] *Center for Photochemical Sciences, Department of Chemistry, Bowling Green State University, Bowling Green, OH 43403*

[f] *Current Affiliation: Department of Engineering Science, Loyola University Chicago, Chicago, IL 60660*

[g] *Current Affiliation: Department of Chemistry, Florida State University, Tallahassee, FL 32306*[fh]*Current*
[h] *Affiliation: Department of Medical Physics, Duke University, Durham, NC 27708*

[i] *Current Affiliation: Syngenta Crop Protection LLC. P.O Box 182200 Greensboro, NC*

* *Corresponding author email: lauren.jarocha@furman.edu*

Number of pages: 8

Number of figures: 5

Number of schemes: 0

Number of tables: 4

Table of Contents

Figure S1: Histogram of bubble size distribution……………………………………………………….3

Table S1: Spin probe loading as a function of size……………………………………………………...3

Figure S2: Comparison between fitting models for EPR spectrum of lipid solutions……………………..4

Table S2: Parameters from fits in Figure S2…………………………………………………………….4

Figure S3: Comparison between fitting EPR spectrum of bubble solutions with and without Heisenberg spin exchange…………………………………………………………………………………....5

Table S3: Parameters from fits in Figure S3…………………………………………………………….5

Figure S4:Comparision between fitting models for EPR spectrum of bubble solutions…………………..6

Table S4: Parameters from fits in Figure S4…………………………………………………………….6

Figure S5: Variation in EPR line shape as a function of key fitting parameters…………………………...7

References……………………………………………………………………………………………….8

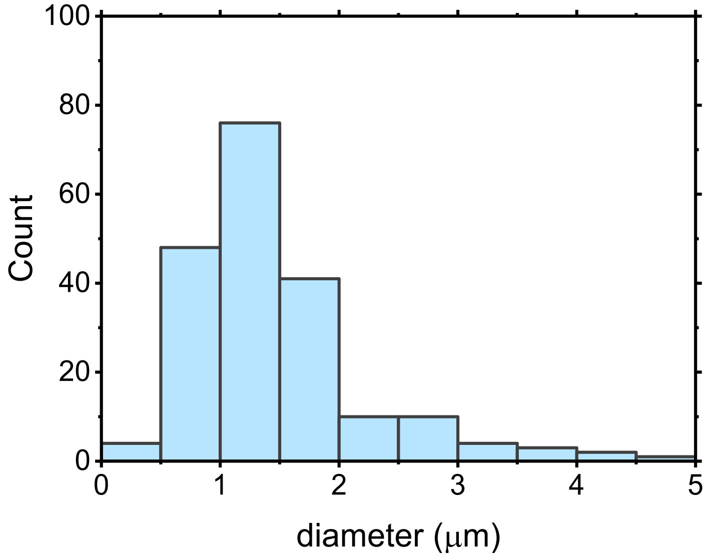

Figure S1: Histogram showing bubble size distribution in the micrograph. A total of 9 bubbles sized above 5 μm are excluded from the figure.

Table S1: Predicted loading of 5DSA as a function of bubble size*

Diameter (μm)	Surface area (μm^2)	number of lipids per bubble	Concentration of bubbles (M)	5DXSA occupation number
**0.1**	0.031	4.8×10^4	2.2×10^{-8}	13
**0.5**	0.76	1.2×10^6	9.0×10^{-10}	334
**0.64**	1.29	2.0×10^6	5.5×10^{-10}	548
**1**	3.14	4.8×10^6	2.2×10^{-10}	1337
**2**	12.5	1.9×10^7	5.6×10^{-11}	5348
**3**	28.3	4.3×10^7	2.5×10^{-11}	12033
**4**	50.3	7.7×10^7	1.4×10^{-11}	21393
**5**	78.5	1.2×10^8	9.0×10^{-12}	33426
**10.3**	333	5.1×10^8	2.1×10^{-12}	141848

* with 3.0×10^{-7} M probe and 1.5 mg/mL total lipid concentration. The surface area per lipid (0.65 nm^2) was estimated based on the aggregation behavior in liposomes.[1] The minimum and maximum diameters found from analysis of Figure 1 are 0.64 μm and 10.3 μm, respectively

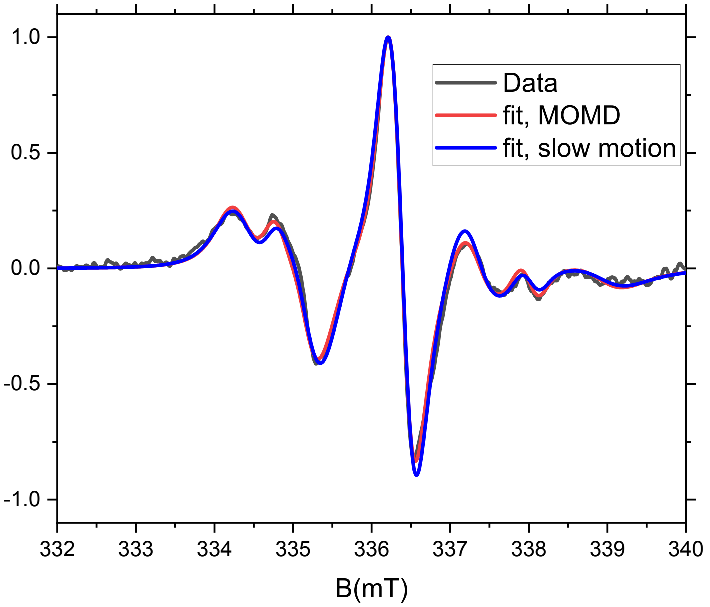

Figure S2: A comparison of fitting the EPR data for 5DSA incorporated into a lipid solution (black) using the MOMD model (red) and or simple anisotropic rotation in the slow motion regime without an ordering potential (blue) Results of the fits are summarized in Table S2.

Table S2: Parameters obtained from EasySpin fits shown in Figure S2.

**Model**	$\tau_\perp$	$\tau_\parallel$	**Azz**	**RMSD**
Slow motion	26.4 ns	4.73 ns	89.9 MHz	30.0
Slow motion, MOMD	21.1 ns	4.65 ns	89.74 MHz	27.45

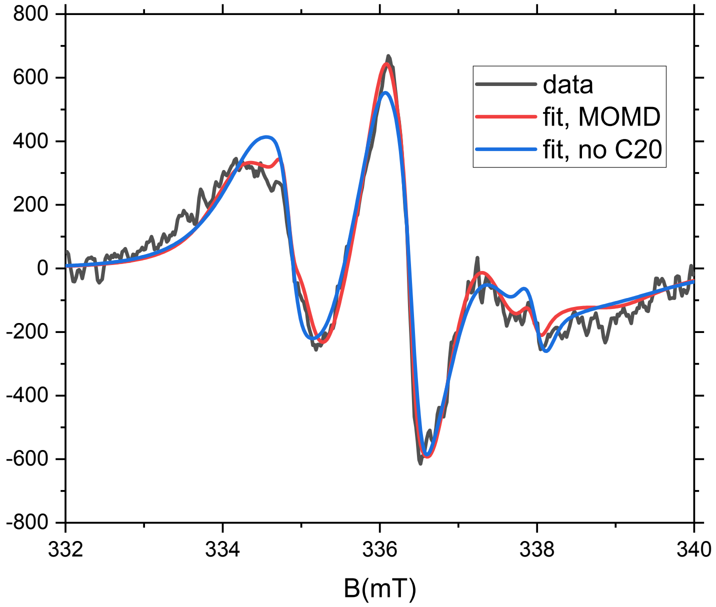

Figure S3: A comparison of fitting the EPR data for 5DSA incorporated into a microbubble solution (black) using the MOMD model (red) and or simple anisotropic rotation in the slow motion regime without an ordering potential (blue) Results of the fits are summarized in Table S3.

Table S3: Parameters obtained from EasySpin fits shown in Figure S3.

**Model**	$\tau_\perp$	$\tau_\parallel$	**Azz**	**C20**	**Exchange**	**RMSD**
Slow motion	5.45 ns	0.46 ns	99.9 MHz	--	11.48 MHz	50.38
Slow motion, MOMD	4.45 ns	0.49 ns	99.9 MHz	2.72	10.48 MHz	38.99

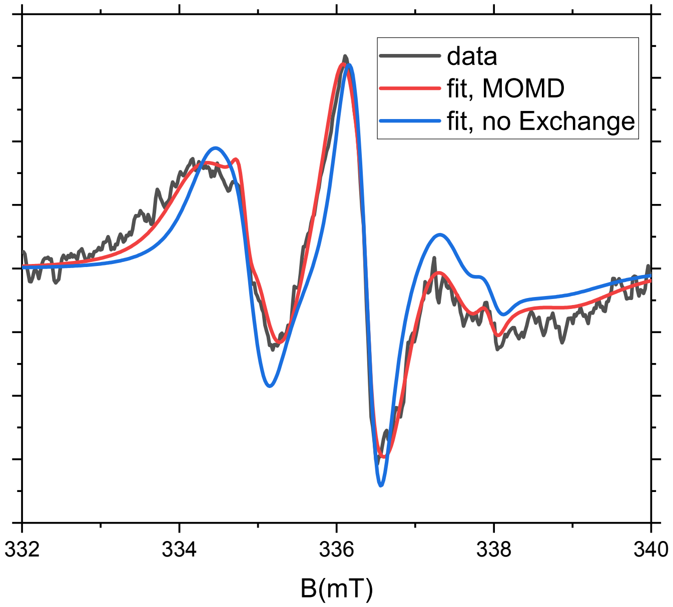

Figure S4: A comparison of fitting the EPR data for 5DSA incorporated into a microbubble solution (black) using the MOMD model including (red) and excluding (blue) Heisenberg spin exchange as a parameter. Results of the fits are summarized in Table S4.

Table S4: Parameters obtained from EasySpin fits shown in Figure S4.

**Model**	$\boldsymbol{\tau}_{\perp}$	$\boldsymbol{\tau}_{\parallel}$	**Azz**	**C20**	**Exchange**	**RMSD**
MOMD, no Exchange	4.64 ns	0.55 ns	97.9 MHz	1.32	0 MHz	86.82
MOMD, Exchange	4.45 ns	0.49 ns	99.9 MHz	2.72	10.48 MHz	38.99

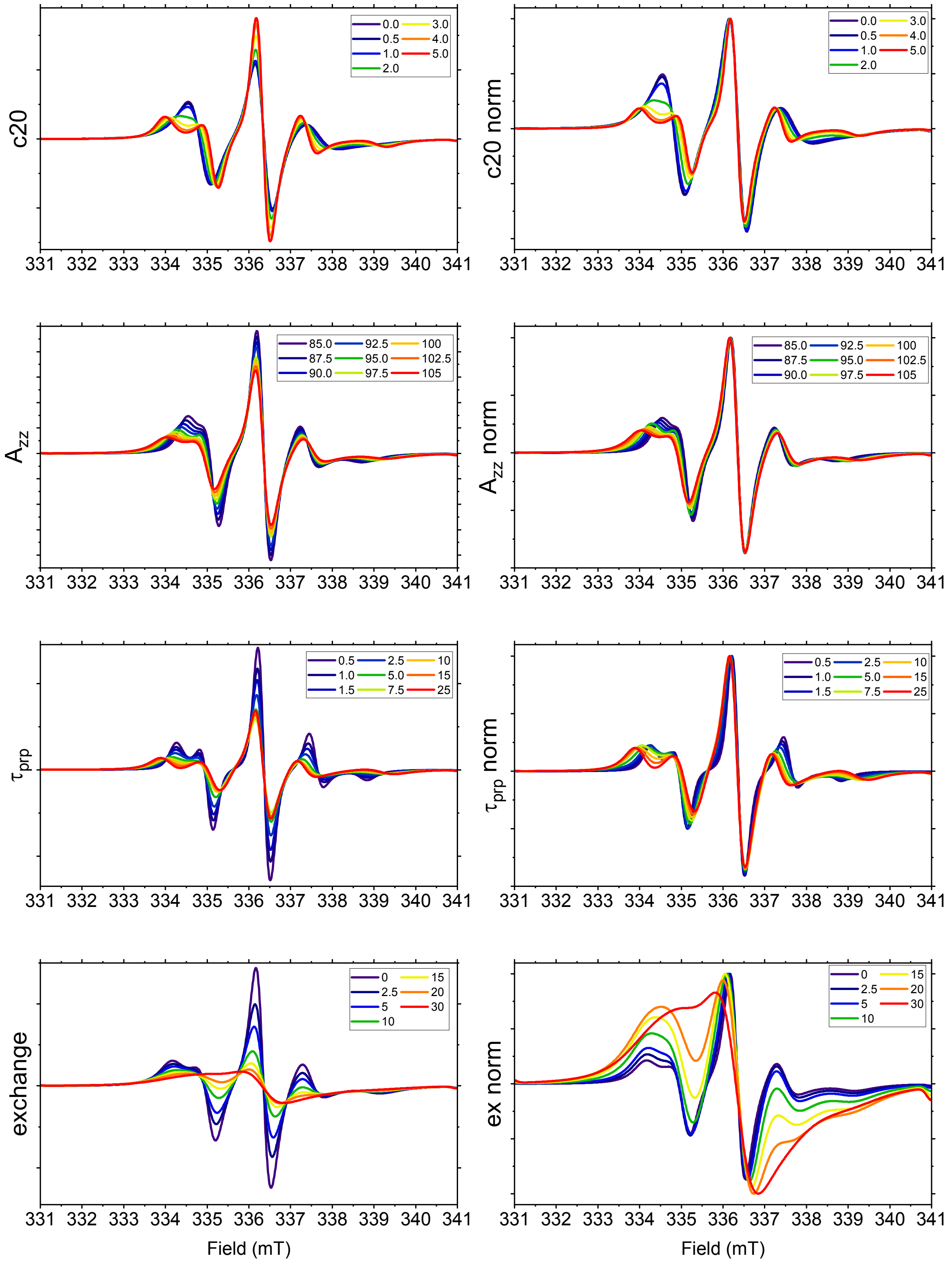

Figure S5. Simulations of the EPR spectrum varying key parameters. Values of all parameters not varied were fixed at the values obtained for the simulations shown in Figure 2 of the text. Units for A_{zz} and Heisenberg spin exchange rate are MHz. Units for $\tau_{\perp}$ are nanoseconds.

References

(1) Takamori, S.; Holt, M.; Stenius, K.; Lemke, E. A.; Grønborg, M.; Riedel, D.; Urlaub, H.; Schenck, S.; Brügger, B.; Ringler, P.; et al. Molecular anatomy of a trafficking organelle. *Cell* **2006**, *127* (4), 831-846. DOI: 10.1016/j.cell.2006.10.030 From NLM.
